# Supplementary material for: Single cell analysis of spondyloarthritis regulatory T cells identifies distinct synovial gene expression patterns and clonal fates
Source: Commun Biol. 2021 Dec 14;4:1395. doi: 10.1038/s42003-021-02931-3 (PMC8671562; doi:10.1038/s42003-021-02931-3)
Supplement: Supplementary file 1 — Supplementary Information [file 42003_2021_2931_MOESM1_ESM.pdf]

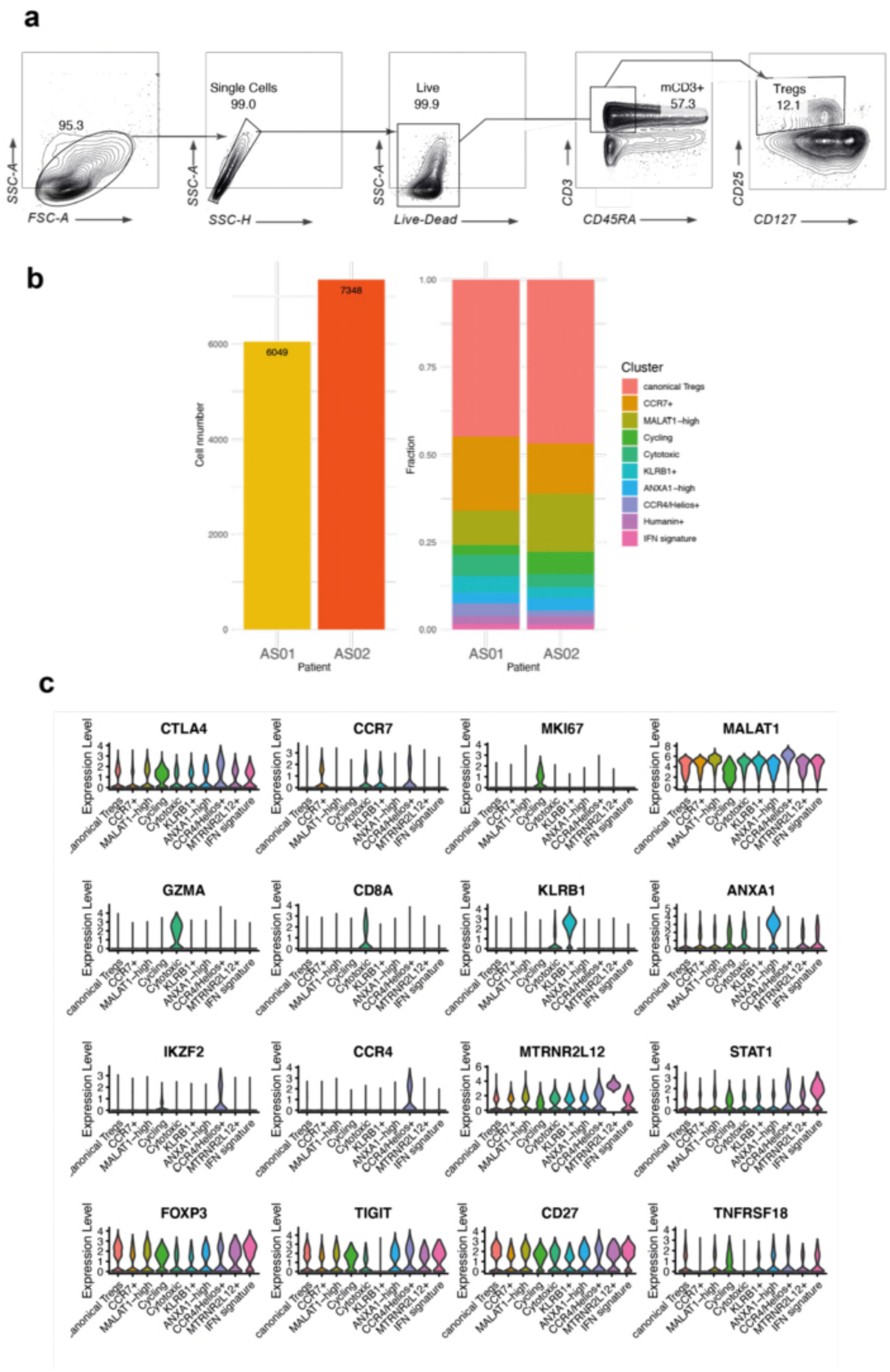

Supplementary Figure 1. Treg sorting strategy, patient contribution to Treg clusters and expression of cluster-characterising genes. (a) Gating strategy used to sort memory Tregs (live CD3$^+$CD45RA$^-$CD25$^+$ CD127^{low}) from peripheral blood and synovial fluid mononuclear cells prior to single cell 10x 5‘ RNA sequencing. **(b)** Patient contribution to the AS dataset and cluster make up in each patient. **(c)** Violin plots showing differential distribution of selected genes (shown normalised expression, log(UMI+1)) used to assist cluster annotation.

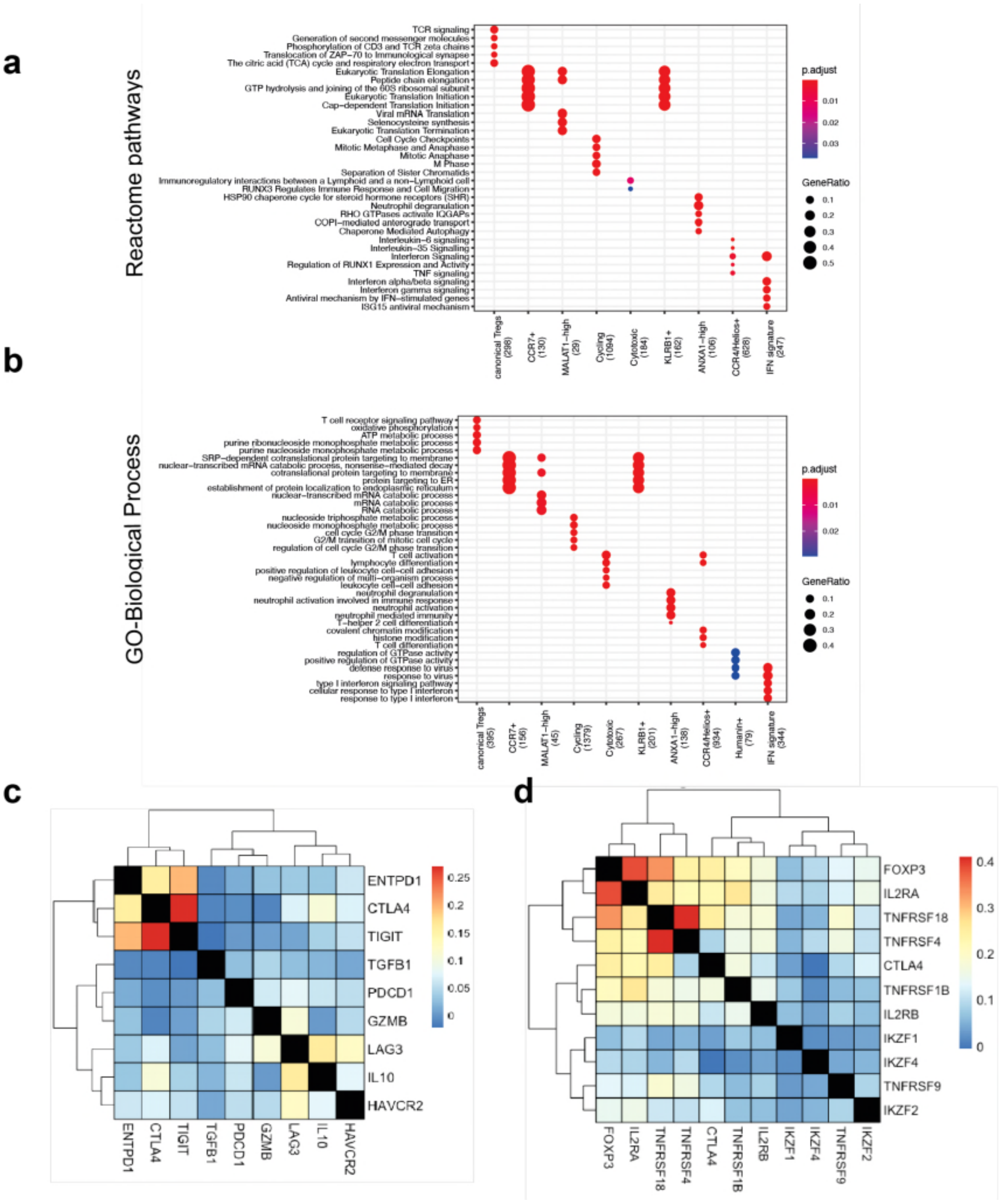

Supplementary Figure 2. Pathway analysis of genes enriched in Treg clusters confirms an interferon-responsive signature cluster; co-expression of key co-inhibitory lineage and transcription factors. a and **b.** Pathway analysis of upregulated genes in each cluster classified by Reactome (**a**) or GO terms Biological Processes (**b**). The color shows the p-value (after Benjamini-Hochberg correction) and the dot size (GeneRatio) represents the overlap between the number of genes upregulated in the cluster and the genes associated with each GO and Reactome term. **c-d** Gene correlation heat maps for selected Treg coinhibitory molecules **(c)** and Treg lineage markers and transcription factors **(d)**. Gene co-expression colored by Spearman's rank correlation coefficient.

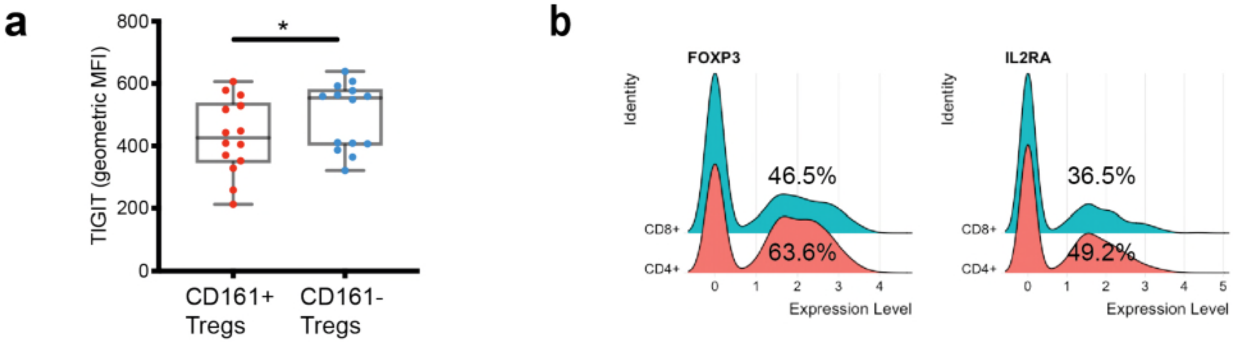

Supplementary Figure 3. Th17-like Tregs are more often TIGIT^{low}, and C8+ Tregs express *FOXP3* and *IL2RA* at levels comparable to CD4+ Tregs.

(a) TIGIT expression (geometric mean of fluorescence intensity) in Tregs from 14 SpA PBMCs (gated on CD4^{+} CD25^{+} CD127^{low}). Box plots show mean and standard error. **(b)** Ridge plots showing normalized expression of *FOXP3* and *IL2RA* in CD4+ and CD8+ Tregs (AS scRNAseq dataset).

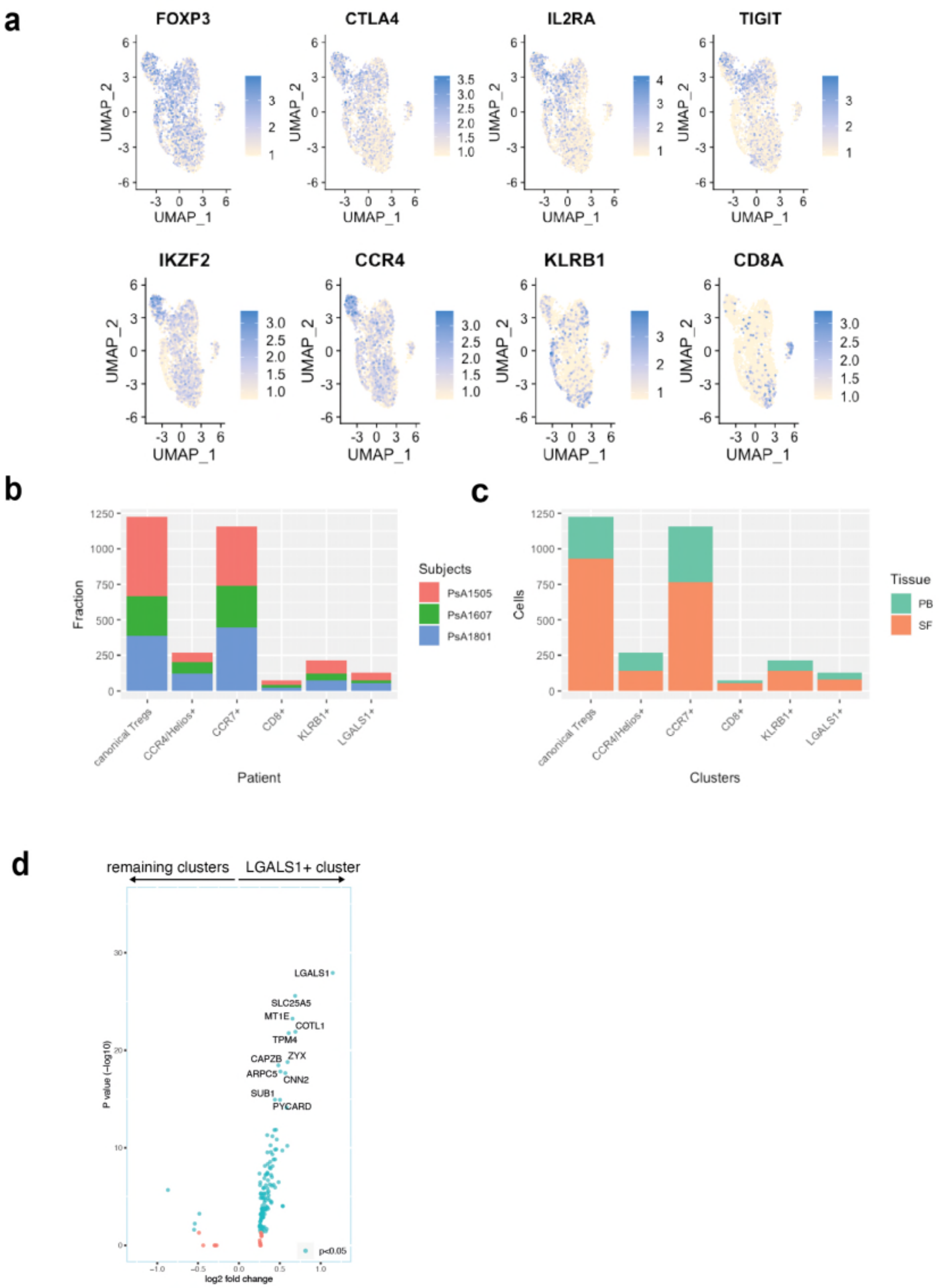

Supplementary Figure 4. Single cell analysis of PsA Tregs confirms cluster identities found in AS. (a) Expression plot of selected genes over the PsA UMAP plot assisting cluster annotation. **(b-c)** Contribution to PsA Treg clusters by patients and by tissue of origin. (**d**) Volcano plot showing genes differentially expressed in the LGALS1+ cluster compared to the rest of PsA Tregs. Blue dots indicate $p<0.05$, Wilcoxon Rank Sum test with Bonferroni correction.

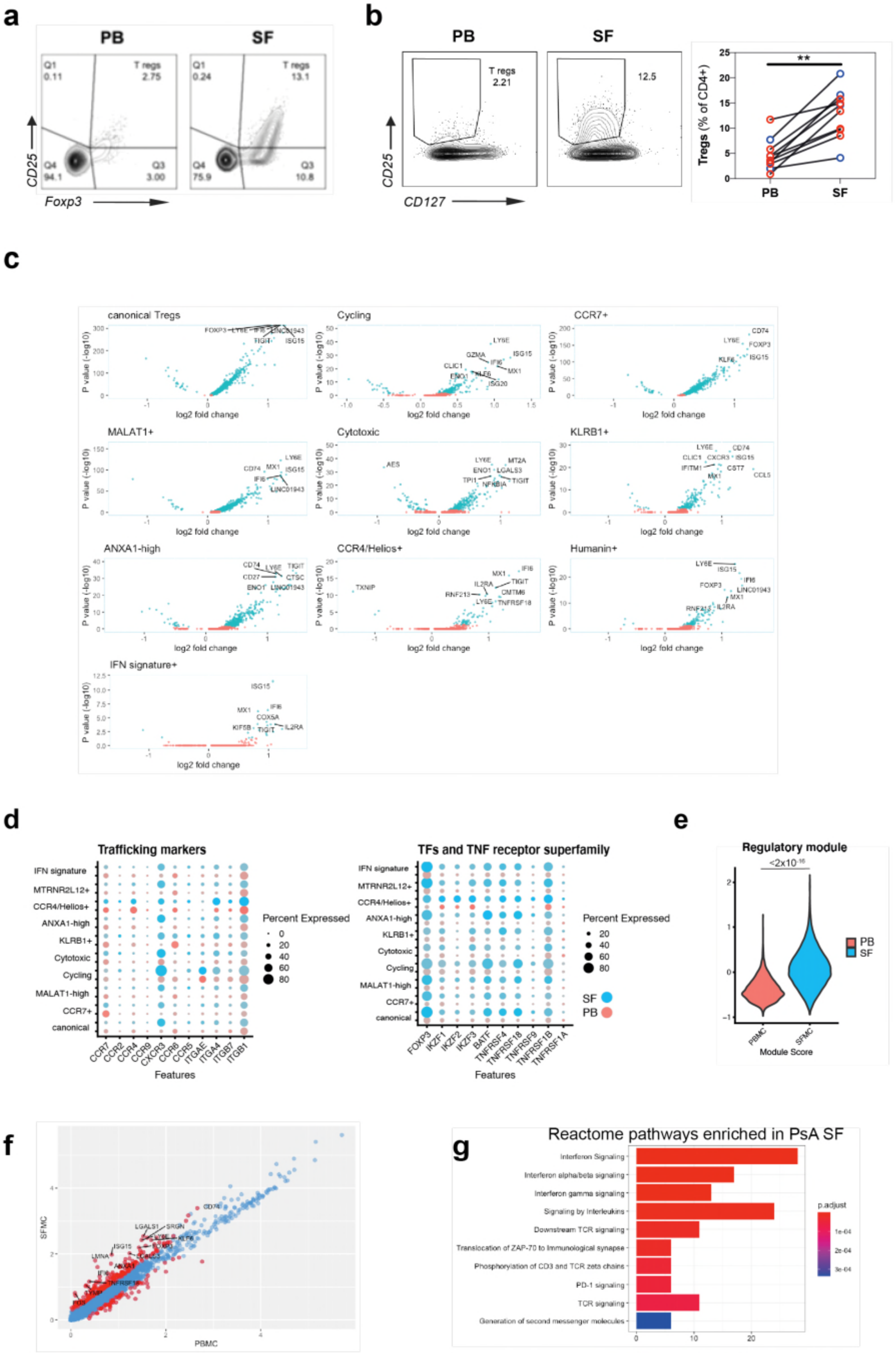
a
PB
SF
Q1 0.11
T regs 2.75
Q4 94.1
Q3 3.00
Q1 0.24
T regs 13.1
Q4 75.9
Q3 10.8
CD25
Foxp3
b
PB
SF
T regs 2.21
12.5
CD25
CD127
Tregs (% of CD4+)
PB
SF
c
canonical Tregs
Cycling
CCR7+
MALAT1+
Cytotoxic
KLRB1+
ANXA1-high
CCR4/Helios+
Humanin+
IFN signature+
P value (-log10)
log2 fold change
d
Trafficking markers
TFs and TNF receptor superfamily
IFN signature
MTRNR2L12+
CCR4/Helios+
ANXA1-high
KLRB1+
Cytotoxic
Cycling
MALAT1-high
CCR7+
canonical
Percent Expressed
SF
PB
Features
CCR7
CCR2
CCR4
CCR9
CXCR3
CCR6
CCR5
ITGAE
ITGA4
ITGB7
ITGB1
FOXP3
IKZF1
IKZF2
IKZF3
BATF
TNFRSF4
TNFRSF18
TNFRSF9
TNFRSF1B
TNFRSF1A
e
Regulatory module
<2x10-16
PB
SF
PBMC
SFMC
Module Score
f
SFMC
PBMC
g
Reactome pathways enriched in PsA SF
Interferon Signaling
Interferon alpha/beta signaling
Interferon gamma signaling
Signaling by Interleukins
Downstream TCR signaling
Translocation of ZAP-70 to Immunological synapse
Phosphorylation of CD3 and TCR zeta chains
PD-1 signaling
TCR signaling
Generation of second messenger molecules
p.adjust

Supplementary Figure 5. Tregs are increased in frequency in SpA synovial fluid and show specific gene expression changes.

(a) Representative flow cytometry plot plots showing CD25 and Foxp3 of CD4+ PB and SF. **(b)** Representative flow cytometry (left panels) and summary data (right panel) showing CD25+ CD127low Tregs from paired SpA PB and SF (gated on CD4+) in PB and SF (n=8, blue dots: AS, red dots: PsA) (right panel). ** p 0.01 (paired t-test). **(c)** Comparison of AS SF and blood (PB) differential gene expression (scRNAseq) for each cluster. Blue plots $p < 0.05$. **(d)** Dot plots showing expression of trafficking markers (left hand panel) and Treg-associated transcription factors and TNF receptor superfamily genes in AS PB (red) and SF (blue dots). **(e)** Regulatory Module Score (obtained from Treg core set in Zemmour *et al*. (9) in PB and SF Tregs (pairwise t-test). **(f)** Normalised logarithmic (log(UMI+1)) expression of all detected genes in PsA PB and SF. Red dots represent genes that are differentially expressed (Wilcoxon rank-sum test with Bonferroni correction). All genes detected in at least 5% of cells in each tissue are computed. **(g)** Genes upregulated in PsA SF compared to the PB grouped into the top 10 enriched Reactome categories. The color represents the p-value (after Benjamini-Hochberg correction) for each enriched Reactome term.

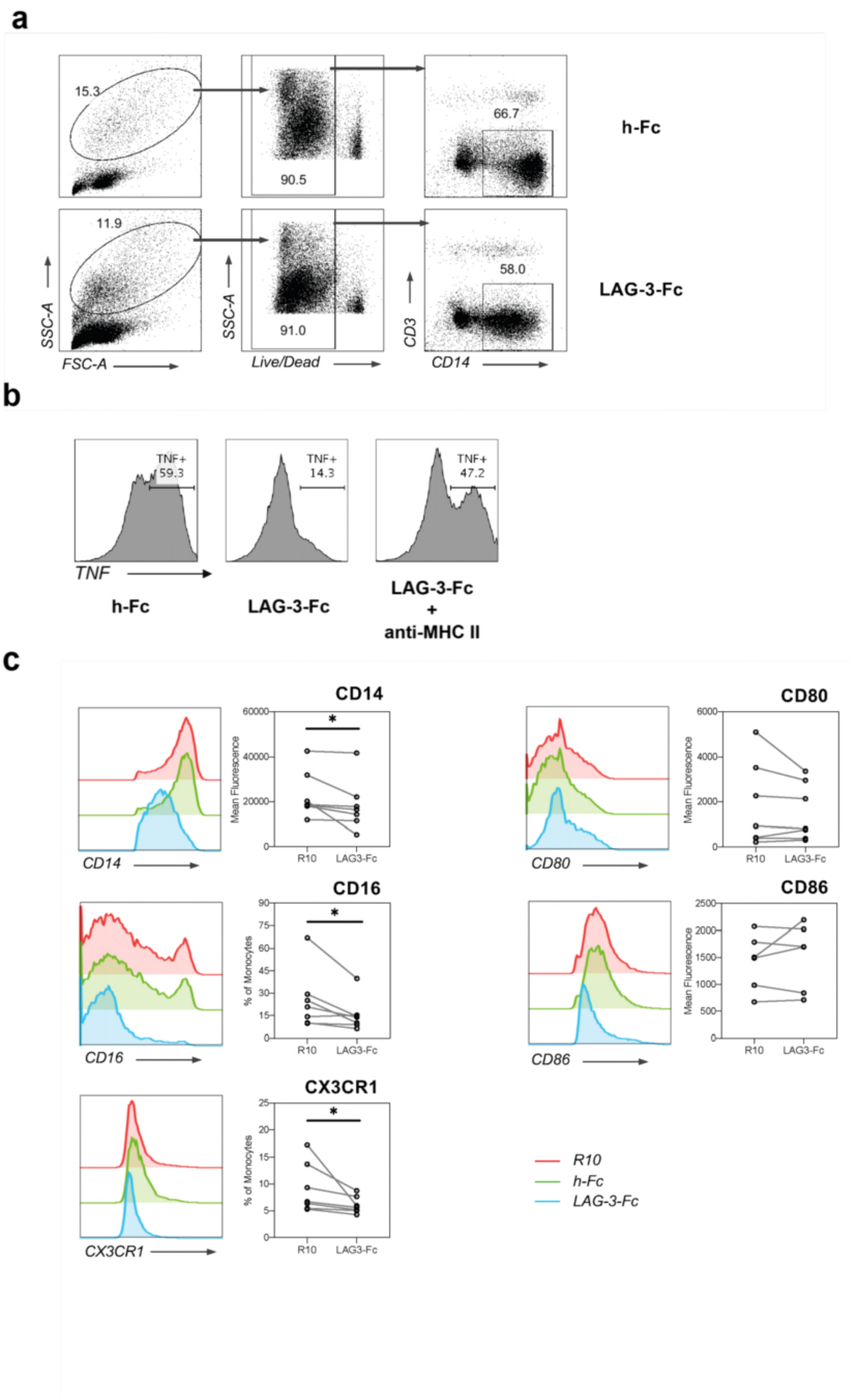
a
15.3
90.5
66.7
h-Fc
11.9
91.0
58.0
LAG-3-Fc
SSC-A
FSC-A
Live/Dead
CD3
CD14
b
TNF+ 59.3
TNF+ 14.3
TNF+ 47.2
TNF
h-Fc
LAG-3-Fc
LAG-3-Fc + anti-MHC II
c
CD14
CD80
CD16
CD86
CX3CR1
Mean Fluorescence
% of Monocytes
R10
LAG3-Fc
R10
h-Fc
LAG-3-Fc

Supplementary Figure 6. Soluble LAG-3 protein modifies monocyte phenotype downregulating selected activation and maturation markers. **(a)** Gating strategy to identify monocytes within PBMCs assay. Downregulation of CD14 expression was observed in the presence of LAG3-Fc. **(b)** Expression of TNF on CD14+ monocytes after overnight culture with LPS and human Fc, LAG-3-Fc with and without blocking anti-MHC II (PBMCs from one AS patient). (c) Selected surface marker expression on monocytes (gated on CD3- CD14+) after overnight culture with LAG-3-Fc fusion protein. For markers with non bimodal distribution, geometric mean of fluorescence intensity was used. n=6 AS PBMC. hFc (human IgG1 Fc protein) was used as control in two of 6 experiments.

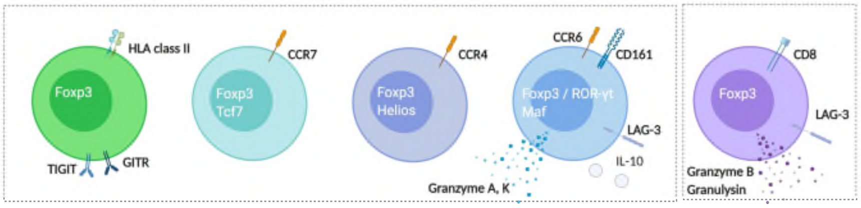

Regulatory mechanism	TIGIT, CTLA-4, CD39	(downregulated, naïve-like phenotype)	CTLA-4	MAF/IL-10, LAG-3, Granzyme K	LAG-3, Granzyme B and K, PD-1
Markers	CD25(hi), HLA-DR, CD27, GITR	CCR7	CCR4	CD161, CCR6, CD6	CD8
Treg core gene expression	high	low	high	low	low
Helios+	+	-	+	- -	+ /-
Synovial fluid changes	TNF receptor superfamily (GITR, TNF-RII), interferon response genes, FOXP3, TIGIT, CCL5				

Supplementary Figure 7. Graphical summary. Distinct functional Treg populations are present in SpA blood and joints. Here shown the most abundant phenotypes identified by our analysis. Figure generated using Biorender.

Patient samples studied with scRNAseq											
	**Age**	**Sex**	**Treatment**	**HLA-B27**	**Peripheral arthritis**	**Skin psoriasis**	**Axial involvement**	**Enthesitis**	**Colitis**	**BASDAI**	**CRP (mg/l)**
**AS**											
**AS01**	41	F	NSAIDs	+	+	-	+	+	-	8.2	31/2
**AS02**	73	F	NSAIDs	+	+	+	+	-	-	N/A	15.0
**PsA**											
**PsA1505**	35	M	NSAIDs	-	+	+	-	-	-	N/A	11.7
**PsA1607**	42	M	None	-	+	+	-	-	-	N/A	8.0
**PsA1801**	54	F	NSAIDs	-	+	-	-	-	-	N/A	9.9

Patient samples studied with flow cytometry						
**Diagnosis**	**Age (Mean ± SD)**	**Sex (M)**	**Treatment**	**HLA-B27 (+)**	**BASDAI (mean) ± SD**	**CRP (mg/l) (mean ± SD)**
PsA 5/8<br>AS 3/8	42.25 ± 12.07	4/8	NSAIDs<br>TNFi (1/8)<br>MTX (1/8)	4/8	6.4 ± 1.8 *	32.3 ± 53.3

Supplementary Table 1. Demographic and clinical characteristics of patients studied with scRNAseq and flow cytometry. * available for AS patients only.

Antibodies used for characterisation of monocyte activation

Antibody	Fluorochrome	Clone	Concentration	Manufacturer
CD14	APC	M5E2	1/50	Biolegend
HLA-DR	PerCP-Cy5.5	L243	1/50	Biolegend
CD80	Brilliant Violet 650	2D10	1/50	Biolegend
CD86	FITC	BU63	1/50	Biolegend
CX3CR1	PE	K0124E1	1/50	Biolegend
CD16	PE/Cy7	3G8	1/50	Biolegend
CD40	Brilliant Violet 421	5C3	1/50	Biolegend
IL-12/23 p40 *	PE	C8.6	1/100	Thermofisher
TNF *	Brilliant Violet 650	MAb11	1/50	Biolegend
Viability Dye	eFluor780		1/500	Thermofisher

Antibodies for FACS sorting for single cell RNAseq

Antibody	Fluorochrome	Clone	Concentration	Manufacturer
CD3	PerCP-Cy5.5	OKT3	1/50	Biolegend
CD4	PE/Dazzle	RPA-T4	1/50	Biolegend
CD8a	PE	RPA-T8	1/50	Biolegend
CD45RA	PE/Dazzle	HI100	1/50	Biolegend
CD25	PE	BC96	1/50	Biolegend
CD127	PE/Cy7	A019D5	1/50	Biolegend
Viability Dye	eFluor520		1/250	Thermofisher

Antibodies used for Treg phenotyping

Antibody	Fluorochrome	Clone	Concentration	Manufacturer
CD3	Brilliant Violet 785	OKT3	1/50	Biolegend
CD4	APC	RPA-T4	1/50	Biolegend
CD25	PE	BC96	1/50	Biolegend
CD127	Brilliant Violet 605	A019D5	1/50	Biolegend
Viability Dye	eFluor780		1/500	Thermofisher
Foxp3 *	Brilliant Violet 421	PCH101	1/50	Invitrogen
CD45RA	PerCP/Cy5.5	HI100	1/50	Biolegend
CTLA-4 *	APC	L3D10	1/50	Biolegend
LAG-3	PE	FAB2319P	1/50	R&D
Granzyme B *	Pe/Dazzle	QA1602	1/50	Biolegend
Granzyme K *	FITC	24C3	1/50	ImmunoTools
CD161	FITC	HP-3G10	Jan-50	Biolegend

Supplementary Table 2. Flow cytometry antibodies used. * antibody used in intracellular staining mix.

Run	Patient	Number of cells	Number of reads (millions)	Mean reads per cell	Median UMI counts per cells	Median genes per cell	Sequencing saturation	Antibody mean reads per cell
1	AS01 PBMC	16507	512.6	31055	3274	1062	74.90%	4946
	AS01 SFMC	18094	501.4	27710	3072	1212	68.20%	4309
2	AS02 PBMC	7959	340.5	42789	2733	1006	86.40%	5201
	AS02 SFMC	16130	584.8	36257	3188	1250	79.70%	3806

Supplementary Table 3. Summary metrics of the two 10x Chromium sequencing runs for the AS samples. Parameters are calculated by the Cell Ranger automated analysis and refer to 5' gene expression and hashing ("antibody reads").

Treg gene core set *(ref 9)*	Th17 gene set
"IL2RA", "IL2RB", "FOXP3", "CTLA4", "IKZF2", "TNFRSF4", "TNFRSF18", "TNFRSF9", "TNFRSF1B"	"RORC", "KLRB1", "CCR6", "IL10", "IL1R1", "IL23R", "MAF", "IL17A", "IL17F", "IL22", "AHR"

Supplementary Table 4. Gene lists used to calculate Module Score for each cluster.
