# Supplementary material for: Single cell analysis of spondyloarthritis regulatory T cells identifies distinct synovial gene expression patterns and clonal fates
Source: Commun Biol. 2021 Dec 14;4:1395. doi: 10.1038/s42003-021-02931-3 (PMC8671562; doi:10.1038/s42003-021-02931-3)
Supplement: Supplementary file 2 — Description of Additional Supplementary Files [file 42003_2021_2931_MOESM2_ESM.pdf]

Description of Additional Supplementary Files

File name: Supplementary Data 1.
Description: AS Differentially expressed genes by cluster.

File name: Supplementary Data 2.
Description: AS Synovial fluid differentially expressed genes.

File name: Supplementary Data 3.
Description: PsA Differentially expressed genes by cluster.

File name: Supplementary Data 4.
Description: SpA (integrated AS/PsA) differentially expressed genes by cluster.

File name: Supplementary Data 5.
Description: Enriched AS TCR clones.

File name: Supplementary Data 6.
Description: Enriched PsA TCR clone.

File name: Supplementary Data 7.
Description: Differentially expressed genes in top 5 synovial fluid clonotypes vs non clonal synovial fluid.

File name: Supplementary Data 8.
Description: LAG-3-mediated monocyte inhibition.

File name: Supplementary Data 9.
Description: Source data to generate figures 1c-d, 2c, 2h, 4b, 5d, 5g, 6c, and 6f.
