# Supplementary material for: Single cell analysis of spondyloarthritis regulatory T cells identifies distinct synovial gene expression patterns and clonal fates
Source: Commun Biol. 2021 Dec 14;4:1395. doi: 10.1038/s42003-021-02931-3 (PMC8671562; doi:10.1038/s42003-021-02931-3)
Supplement: Supplementary file 12 — Reporting Summary [file 42003_2021_2931_MOESM12_ESM.pdf]

nature research

Corresponding author(s): Davide Simone

Last updated by author(s): Nov 10, 2021

Reporting Summary

Nature Research wishes to improve the reproducibility of the work that we publish. This form provides structure for consistency and transparency in reporting. For further information on Nature Research policies, see our Editorial Policies and the Editorial Policy Checklist.

Statistics

For all statistical analyses, confirm that the following items are present in the figure legend, table legend, main text, or Methods section.

n/a	Confirmed	
☐	☒	The exact sample size (*n*) for each experimental group/condition, given as a discrete number and unit of measurement
☐	☒	A statement on whether measurements were taken from distinct samples or whether the same sample was measured repeatedly
☐	☒	The statistical test(s) used AND whether they are one- or two-sided<br>*Only common tests should be described solely by name; describe more complex techniques in the Methods section.*
☐	☒	A description of all covariates tested
☐	☒	A description of any assumptions or corrections, such as tests of normality and adjustment for multiple comparisons
☐	☒	A full description of the statistical parameters including central tendency (e.g. means) or other basic estimates (e.g. regression coefficient) AND variation (e.g. standard deviation) or associated estimates of uncertainty (e.g. confidence intervals)
☐	☒	For null hypothesis testing, the test statistic (e.g. *F*, *t*, *r*) with confidence intervals, effect sizes, degrees of freedom and *P* value noted<br>*Give P values as exact values whenever suitable.*
☒	☐	For Bayesian analysis, information on the choice of priors and Markov chain Monte Carlo settings
☐	☒	For hierarchical and complex designs, identification of the appropriate level for tests and full reporting of outcomes
☒	☐	Estimates of effect sizes (e.g. Cohen's *d*, Pearson's *r*), indicating how they were calculated

Our web collection on statistics for biologists contains articles on many of the points above.

Software and code

Policy information about availability of computer code

Data collection	CellRanger (v3.1)<br>TraCeR<br>HighVQuest<br>kallisto v0.43.1
Data analysis	Seurat v 3.1.4<br>GLIPH v 1.0<br>VDJtools 1.1.8

For manuscripts utilizing custom algorithms or software that are central to the research but not yet described in published literature, software must be made available to editors and reviewers. We strongly encourage code deposition in a community repository (e.g. GitHub). See the Nature Research guidelines for submitting code & software for further information.

Data

Policy information about availability of data

All manuscripts must include a data availability statement. This statement should provide the following information, where applicable:

- Accession codes, unique identifiers, or web links for publicly available datasets
- A list of figures that have associated raw data
- A description of any restrictions on data availability

The scRNA-seq data and scTCR-seq data sets included in this paper will be deposited in ArrayExpress with the accession code E-MTAB-10948. Source data used for the generation of figures 4A, 5A, 5C is provided with this paper. Sequencing data from the PsA dataset15 have been deposited in ArrayExpress with the accession code E-MTAB-9492 and in the European Genome-phenome Archive (EGA) with accession code EGAS00001002104.

Field-specific reporting

Please select the one below that is the best fit for your research. If you are not sure, read the appropriate sections before making your selection.

☒ Life sciences ☐ Behavioural & social sciences ☐ Ecological, evolutionary & environmental sciences

For a reference copy of the document with all sections, see nature.com/documents/nr-reporting-summary-flat.pdf

Life sciences study design

All studies must disclose on these points even when the disclosure is negative.

Sample size	No sample size calculation was performed for RNA sequencing experiments. The number of cells sequenced was based on previous published data-sets.
Data exclusions	In the single cell RNA sequencing experiment, after cell-containing droplets were identified, gene-expression matrices were first filtered to remove cells having >10% mitochondrial gene transcripts, <250 or >4,000 genes expressed or >25,000 UMI (Unique Molecular Identifiers). The Seurat demultiplexing function ("HTODemux", with a threshold set at the 99th quantile of the negative binomial distribution for the oligo) was then used to demultiplex the hashing library in order to identify Tregs and to remove doublets and other non Treg cells. Cells were further filtered to exclude cells not expressing any transcripts from CD3 complex-associated genes (CD3E, CD3D, CD3G) and TCR multiplets (defined as cells with greater than 1 TCR beta chain or greater than 2 TCR alpha chains).<br>To further remove any CD14+ cells or multiplets which may have escaped exclusion by cell sorting, a preliminary round of dataset integration, dimensionality reduction and cell clustering as described below was used to identify cells belonging to CD14+ clusters. These cells, along with any additional cells expressing CD14, were then excluded from the input used in generating a final integrated dataset.
Replication	Gene expression validation of 10x 5' data was done in a second dataset of a related condition, obtained from a dataset generated previously by our group (Penkava et al., 2020)
Randomization	N/A
Blinding	No blinding was carried out in this study

Reporting for specific materials, systems and methods

We require information from authors about some types of materials, experimental systems and methods used in many studies. Here, indicate whether each material, system or method listed is relevant to your study. If you are not sure if a list item applies to your research, read the appropriate section before selecting a response.

Materials & experimental systems

n/a	Involved in the study	
☐	☒	Antibodies
☒	☐	Eukaryotic cell lines
☒	☐	Palaeontology and archaeology
☒	☐	Animals and other organisms
☐	☒	Human research participants
☒	☐	Clinical data
☒	☐	Dual use research of concern

Methods

n/a	Involved in the study	
☒	☐	ChIP-seq
☐	☒	Flow cytometry
☒	☐	MRI-based neuroimaging

Antibodies

Antibodies used	Listed in Supplementary Table 2.
Validation	No new antibodies were generated for this study, only commercially available antibodies

Human research participants

Policy information about studies involving human research participants

Population characteristics	Patients with AS and PsA were recruited during routine clinical care following written informed consent in accordance with the protocol approved by the South Central – Oxford C Research Ethics Committee (IFIA, Immune Function in Inflammatory Arthritis: ethics reference 06/Q1606/139). All patients (clinical characteristics shown in Supplementary Table 1) fulfilled the disease classification criteria (respectively ASAS and CASPAR), and were naïve to biologic disease-modifying antirheumatic drugs (DMARD) and not on any conventional DMARD at the time of the sample. All patients with AS were HLA-B27 positive with evidence of active axial and peripheral joint involvement, and both were of female sex. Patients with PsA had large joint

	peripheral oligoarthritis although none were HLA-B27 positive. Synovial fluid samples were obtained during knee joint aspiration performed for therapeutic reasons.
Recruitment	Patients fulfilling criteria for the study were recruited sequentially in the Oxford Rheumatology Hospitals rheumatology clinic
Ethics oversight	Oxford Research Ethics committee (Ethics reference number 06/Q1606/139)

Note that full information on the approval of the study protocol must also be provided in the manuscript.

Flow Cytometry

Plots

Confirm that:

☒ The axis labels state the marker and fluorochrome used (e.g. CD4-FITC).

☒ The axis scales are clearly visible. Include numbers along axes only for bottom left plot of group (a 'group' is an analysis of identical markers).

☒ All plots are contour plots with outliers or pseudocolor plots.

☒ A numerical value for number of cells or percentage (with statistics) is provided.

Methodology

Sample preparation	After isolation by density centrifugation, PBMC and SFMC were immediately stained with fluorescently conjugated antibodies in RNAse-free PBS, 2 mM EDTA and then FACS-sorted prior to droplet-based single-cell RNA sequencing. AS samples were stained with the following antibodies: CD3-PerCP-Cy5.5 (OKT3), CD8a-PE (RPA-T4), CD45RA-PE/Dazzle (HI100), CD25-PE (BC96), CD127-PE/Cy7 (A019D5) (all from Biolegend, and used at 1:50 dilution) and Fixable Viability Dye eFluor520 (eBioscience, dilution 1:250) to exclude dead cells. Cells were sorted on a Sony SH800Z and collected in a collection buffer (Phenol Red-ve RPMI + 4% Bovine Serum Albumin + Hepes 25mM). After sorting, cells were stained separately with Fc blocker (concentration 1:20) (TruStain FcX, Biolegend), rested for 15 minutes, then washed, then resuspended in buffer to be further stained with with the oligo-tagged TotalSeq™-C0251 Hashtag antibody. Cells were again washed twice with FACS buffer then kept on ice until loaded onto the Chromium controller. For sample AS02, PBMC and SFMC were not processed fresh but thawed after being cryopreserved in liquid nitrogen.
Instrument	A Sony SH800Z were used for cell sorting
Software	Flowjo version 10
Cell population abundance	Purity was always greater than 99%
Gating strategy	Memory Tregs were sorted as in Supplementary Fig. 1A (CD45RA-(negative) CD3+ CD25+ CD127low).

☒ Tick this box to confirm that a figure exemplifying the gating strategy is provided in the Supplementary Information.
